# Correction for Pfannenstiel et al., “Identification of a series of pyrrolo-pyrimidine based SARS-CoV-2 Mac1 inhibitors that repress coronavirus replication”

**DOI:** 10.1128/mbio.00865-26

**Published:** 2026-04-14

**Authors:** Jessica J. Pfannenstiel, Men Thi Hoai Duong, Daniel Cluff, Lavinia M. Sherrill, Iain Colquhoun, Gabrielle Cadoux, Devyn Thorne, Johan Pääkkönen, Nathaniel F. Schemmel, Joseph O’Connor, Pradtahna Saenjamsai, Mei Feng, Srivatsan Parthasarathy, Michael J. Hageman, David K. Johnson, Anuradha Roy, Lari Lehtiö, Dana V. Ferraris, Anthony R. Fehr

## AUTHOR CORRECTION

Vol. 16, no. 6, e03865-24, 2025, https://doi.org/10.1128/mbio.03865-24. Acknowledgements, sentence 2: “and an undergraduate research scholarship provided to NFS from the NIH funded Kansas IDeA Network of Biomedical Research Excellence (K-INBRE) (P20GM103418)” should be removed.

